# *Salmonella* Adhesion, Invasion and Cellular Immune Responses Are Differentially Affected by Iron Concentrations in a Combined *In Vitro* Gut Fermentation-Cell Model

**DOI:** 10.1371/journal.pone.0093549

**Published:** 2014-03-27

**Authors:** Alexandra Dostal, Mélanie Gagnon, Christophe Chassard, Michael Bruce Zimmermann, Liam O'Mahony, Christophe Lacroix

**Affiliations:** 1 Laboratory of Food Biotechnology, Institute of Food, Nutrition and Health, ETH Zurich, Zurich, Switzerland; 2 Laboratory of Human Nutrition, Institute of Food, Nutrition and Health, ETH Zurich, Zurich, Switzerland; 3 Swiss Institute of Allergy and Asthma Research, University of Zurich, Davos, Switzerland; Facultad de Medicina, Uruguay

## Abstract

In regions with a high infectious disease burden, concerns have been raised about the safety of iron supplementation because higher iron concentrations in the gut lumen may increase risk of enteropathogen infection. The aim of this study was to investigate interactions of the enteropathogen *Salmonella enterica* ssp. *enterica* Typhimurium with intestinal cells under different iron concentrations encountered in the gut lumen during iron deficiency and supplementation using an *in vitro* colonic fermentation system inoculated with immobilized child gut microbiota combined with Caco-2/HT29-MTX co-culture monolayers. Colonic fermentation effluents obtained during normal, low (chelation by 2,2'-dipyridyl) and high iron (26.5 mg iron/L) fermentation conditions containing *Salmonella* or pure *Salmonella* cultures with similar iron conditions were applied to cellular monolayers. *Salmonella* adhesion and invasion capacity, cellular integrity and immune response were assessed. Under high iron conditions in pure culture, *Salmonella* adhesion was 8-fold increased compared to normal iron conditions while invasion was not affected leading to decreased invasion efficiency (−86%). Moreover, cellular cytokines IL-1β, IL-6, IL-8 and TNF-α secretion as well as NF-κB activation in THP-1 cells were attenuated under high iron conditions. Low iron conditions in pure culture increased *Salmonella* invasion correlating with an increase in IL-8 release. In fermentation effluents, *Salmonella* adhesion was 12-fold and invasion was 428-fold reduced compared to pure culture. *Salmonella* in high iron fermentation effluents had decreased invasion efficiency (−77.1%) and cellular TNF-α release compared to normal iron effluent. The presence of commensal microbiota and bacterial metabolites in fermentation effluents reduced adhesion and invasion of *Salmonella* compared to pure culture highlighting the importance of the gut microbiota as a barrier during pathogen invasion. High iron concentrations as encountered in the gut lumen during iron supplementation attenuated *Salmonella* invasion efficiency and cellular immune response suggesting that high iron concentrations alone may not lead to an increased *Salmonella* invasion.

## Introduction

Infections with enteric pathogens and the resulting diarrhea are a cause of morbidity worldwide and especially in developing countries [1]. During intestinal infection *Salmonella enterica* ssp. *enterica* Typhimurium (*S.* Typhimurium), a non-typhoidal strain causing severe diarrhea, has to overcome several barriers implemented by the commensal microbiota as well as host defense mechanisms. One of these mechanisms of the host is to limit essential nutrients, such as iron (Fe), to the pathogen on the intestinal epithelial surface and intracellularly [2], [3], [4]. The Fe-limiting activities of the host during inflammation are now regarded as part of the innate immune system and involve the production of bacterial siderophore sequestering molecules (lipocalin) and the limitation of intracellular Fe mediated by inflammatory cytokines [5]. *S.* Typhimurium has a requirement for Fe and therefore has developed strategies to overcome Fe limitation, such as the production of the Fe chelator salmochelin [6] and the activation of virulence mechanisms under different Fe conditions. In fact, about 7% of the *Salmonella* genome is regulated by Fe [7] and growth is promoted at high Fe conditions [8]. Acquisition of Fe during infection seems to be crucial to develop full virulence [9]. It has been reported that the type III secretion system, which mediates the invasion into the intestinal epithelium, is stimulated under elevated Fe conditions while under low Fe availability the expression of the type III secretion system is decreased [8], [10], [11]. Indeed, *in vitro* invasion assays of *Salmonella* into epithelial cell layers have shown an increased invasion capacity in elevated Fe conditions [12], [13]. However, under very high Fe conditions, invasion seemed to be decreased while adhesion of *Salmonella* was increased [13]. Other studies have shown that host Fe status can determine the severity of bacterial infection. For example in mice, treatment with the Fe chelator deferoxamine exacerbated *Salmonella* infection [14] but also patients suffering from Fe overload are reported to be more susceptible to infections [15].

When colonizing the gut, *Salmonella* has to overcome also barrier mechanisms implemented by the commensal gut microbiota [16]. Not only nutrient depletion by the gut microbiota in the intestinal lumen, but also production of metabolites such as short chain fatty acids (SCFA) may have detrimental effects on *Salmonella* growth. It has been shown, that propionate and especially butyrate decreases *Salmonella* invasion *in vitro* [17] by down regulating the pathogenicity island 1 gene expression [18]. On the other hand, acetate increased *Salmonella* invasion [17]. This concept was also proven *in vivo* in chickens, where the feed additive of butyrate decreased and acetate increased *Salmonella* counts in caecum [19]. However, a recent study showed that acetate produced by bifidobacteria may also protect from enteropathogenic *E. coli* [20]. Moreover, effects of commensal gut bacteria on the invasion capacity of *Salmonella* have been observed. Lactobacilli and bifidobacteria, often used as probiotics, can attenuate *Salmonella* invasion by strengthening the epithelial barrier functions [21], [22], [23], [24], [25], [26]. The gut microbiota might also stimulate immune defenses of the host against pathogens [16], [26], [27].

Fe is not only essential for pathogens but also for the host. Fe deficiency is one of the most common nutritional deficiencies and affects around 2 billion people worldwide, especially in regions with plant based diets low in bioavailable Fe, such as in many developing countries [28], [29]. A common strategy to correct Fe deficiency is Fe supplementation leading to very high Fe concentrations in the gut lumen. However, Fe supplementation has been associated with an increased risk for infectious disease in regions with high prevalence of malaria, tuberculosis and enteric pathogens [30], [31], [32]. Moreover, both Fe deficiency and Fe supplementation have been shown to alter the commensal bacterial ecosystem in the gut and impact the gut microbiota metabolic activity [33], [34], [35], [36]. These changes in the gut microbiota due to Fe availability might negatively impact barrier functions of the gut microbiota and provide less protective properties to the epithelial layer against enteropathogens.

Considering the importance of Fe for *Salmonella* virulence, for host immunity and for the commensal gut microbiota, the highly variable amounts of Fe in the intestinal lumen encountered during Fe deficiency and high-dose Fe supplementation could modulate the adhesion and invasion pattern of *S*. Typhimurium. Therefore, the aim of this study was to investigate the impact of Fe availability in the gut lumen on the adhesion and invasion of *S*. Typhimurium alone or in a complex bacterial environment using a combined *in vitro* gut fermentation-cell model. *Salmonella* single bacterial cultures and effluents from a colonic *in vitro* fermentation study investigating the impact of different dietary Fe concentrations on the gut microbiota [36] containing *Salmonella* were tested in a co-culture model of Caco-2/HT29-MTX cells for adhesion and invasion. The cellular co-culture model combined two major cell phenotypes found in the intestine, Fe absorptive Caco-2 cells and mucus secreting HT29-MTX cells, to provide an epithelial monolayer covered with mucus which better mimics the situation *in vivo* [37], [38], [39]. Besides *Salmonella* adhesion and invasion, epithelial cell layer integrity, cytokine profiles and immune responses of Caco-2/HT29-MTX co-cultures in contact with peripheral blood monocytic cells (PBMC) were determined during infection with *Salmonella* under differing Fe conditions.

## Materials and Methods

### Bacterial strain and growth conditions

The *Salmonella enterica* ssp. *enterica* serovar Typhimurium N-15 (*S*. Typhimurium N-15) isolate used in this study originated from an infected patient and was obtained from the National Center for Enteropathogenic Bacteria (NENT, Zurich, Switzerland). Previous studies with this strain have shown high adhesion and invasion capacities [22]. *S*. Typhimurium N-15 was cultivated in tryptic soy broth (TSB, Difco, Basel, Switzerland) at 37°C.

### Continuous colonic fermentation effluents with a complex gut microbiota

Fermentation effluents containing a complex gut microbiota were produced in an *in vitro* continuous colonic fermentation system using immobilized child fecal microbiota mimicking proximal colon conditions of a child aimed to investigate the impact of Fe deficiency and Fe supplementation on child gut microbiota, as previously described [36]. Briefly, two reactors containing immobilized gut microbiota of the same child were continuously operated in parallel for 70 days and different Fe conditions in the nutritive medium were applied for 10 days each until a pseudo-steady state was reached in the reactors (7 treatment periods). Fermentation effluents were collected during the last 3 days of each fermentation period showing a stable microbiota, shock frozen in liquid nitrogen immediately after sampling, and stored at -80°C until further use. In this study, fermentation effluents under normal Fe conditions (normal Fe effluent, 8.13±1.8 mg Fe/L), high Fe conditions encountered in chyme of the intestinal lumen of a child during Fe supplementation (high Fe effluent, 26.5±2.2 mg Fe/L) and low Fe conditions (low Fe effluent, Fe chelated by the addition of 150 μM 2,2′-dipyridyl (Sigma-Aldrich, Buchs, Switzerland)) were used to assess the invasion capacity of *S*. Typhimurium N-15 in the presence of a complex gut microbiota and bacterial metabolites. Microbial composition of fermentation effluents was analyzed by qPCR and metabolic activity by HPLC and detailed results are reported in a previous publication [36].

### Caco-2/HT29-MTX co-culture

A co-culture of 75% Caco-2 absorptive cells and 25% mucus-secreting HT29-MTX cells was used for all assays [37], [38]. Caco-2 cells were obtained from Leibnitz Institute DSMZ (German Collection of Microorganisms and Cell Cultures, Braunschweig, Germany), used between passages 8 to 20 and were routinely maintained in Dulbecco's Modified Eagle medium (DMEM, Invitrogen, Basel, Switzerland) supplemented with 20% fetal bovine serum (FBS, Invitrogen), 1% antibiotics (penicillin/streptomycin, Invitrogen) and 1% non-essential aminoacids (NEAA, Invitrogen) at 37°C in a humidified incubator (5% CO_2_). The mucus-secreting intestinal cells HT29-MTX were kindly provided by T. Lesuffleur (INSERM, Lille, France) [40]. HT29-MTX cells (passages 5 to 10) were routinely maintained in DMEM Glutamax (Invitrogen) supplemented with 10% FBS and 1% antibiotics (penicillin/streptomycin, Invitrogen) at 37°C in a humidified incubator (5% CO_2_). Caco-2 cells and HT29-MTX cells were prepared as previously described [37], seeded in a 75∶25 ratio in 24-well tissue culture plates (Bioswisstec, Schaffhausen, Switzerland) at a final concentration of 50000 cells/cm^2^ and cultivated for 16 days to reach differentiation. The co-culture was maintained in DMEM Glutamax with 10% FBS (Invitrogen) and 1% antibiotics (streptomycin/penicillin, Invitrogen) at 37°C in a humidified incubator (5% CO_2_) with medium exchanged every 2 days.

### Adhesion and invasion assays

For the investigation of *S*. Typhimurium N-15 adhesion and invasion capacity, a gentamicin-based assay previously described by Gagnon *et al*. was used [39]. After the epithelial co-culture had reached full differentiation (16 days) the medium was exchanged to antibiotics-free medium for 24 hours. An overnight culture of *S*. Typhimurium N-15 was inoculated at 1% into fresh TSB and grown at 37°C for 6 h to reach a bacterial cell density of approximately 10^8^ colony forming units (cfu) per mL. Bacterial cells were centrifuged and washed twice with PBS before 1∶10 dilution with DMEM Glutamax with different Fe concentrations or fermentation effluents (normal Fe effluents, low Fe effluents and high Fe effluents, thawed on ice) to reach a *S*. Typhimurium N-15 concentration of 10^7^ cfu/mL resulting in a multiplicity of infection of approximately 100. For invasion in DMEM Glutamax, the medium was adapted to mimic normal Fe conditions (Normal Fe DMEM), obtained by using DMEM Glutamax without the addition of FBS or antibiotics, low Fe conditions (low Fe DMEM), obtained by adding 150 μM Fe chelator 2,2′-dipyridyl (Sigma-Aldrich) to DMEM Glutamax, and high Fe conditions (high Fe DMEM), obtained by adding 14 mg Fe/L (250 μM Fe) as FeSO_4_ to DMEM Glutamax. Adhesion and invasion assays were performed as described previously [22], [39]. Briefly, 500 μL effluent or DMEM Glutamax containing *S*. Typhimurium N-15 were added per well of co-culture and after incubation for 60 min at 37°C, supernatants were removed and cells were washed twice with PBS. After the addition of 250 μL DMEM Glutamax containing 150 μg/mL gentamicin (Sigma-Aldrich) to kill extracellular *S*. Typhimurium N-15 for 45 min, cells were disrupted with 0.1% Triton X-100. Serial dilutions of the disrupted cell suspension were then plated on selective CHROM-Agar (Becton Dickinson, Allschwil, Switzerland) to enumerate invaded bacteria. To evaluate the number of adhered *S*. Typhimurium N-15, the same protocol was used but without the gentamicin treatment. Adhesion and invasion rates were calculated as percentages of adhered or invaded *S*. Typhimurium N-15 to initially applied numbers. Invasion efficiency was calculated as percentage ratio of invaded to adhered bacteria. For the adhesion and invasion assays in DMEM Glutamax with different Fe concentrations (each conditions applied in triplicate), 3 independent experiments were performed using different Caco-2/HT29-MTX passages each (n = 3). For the adhesion and invasion assays in fermentation effluents, 2 independent experiments were performed using 5 different Caco-2/HT29-MTX passages (n = 5).

### Cell cytotoxicity assay

Lactate dehydrogenase (LDH), a marker for cell toxicity, release into the cellular supernatant was evaluated during invasion in fermentation effluent. In short, after the 60 min invasion of *S*. Typhimurium N-15 in fermentation effluent with different Fe concentrations, supernatant was collected and centrifuged. LDH concentration in the supernatant was then evaluated using the Cytotox assay (Promega, Madison, WI, USA). LDH release was calculated as percentage of maximum LDH release during invasion of *S*. Typhimurium N-15 under normal Fe conditions in DMEM Glutamax. This assay was performed during invasion in 3 different passages of Caco-2/HT29-MTX (n = 3).

### TER assay

Transepithelial electrical resistance (TER) was measured for the evaluation of tight junction disruption during *S*. Typhimurium N-15 invasion under different Fe concentrations with and without a commensal microbiota. Caco-2/HT29-MTX co-cultures were seeded on cell culture filter membranes (0.45 μm) in 24-well plates (Millipore, Zug, Switzerland) as described above. After 16 days of cultivation, monolayers were washed once with PBS before 500 μL of DMEM Glutamax or fermentation effluent with different Fe concentrations containing *S*. Typhimurium N-15 (10^7^ cfu/mL; as described in adhesion and invasion assays) were added to the apical compartment. DMEM Glutamax with the corresponding Fe concentration was added in the basolateral compartment. TER measurement was done immediately after the addition of *S*. Typhimurium N-15 (t = 0 h) and after 1, 2, 4 and 6 h of incubation (37°C, 5% CO_2_). Changes in TER over time were calculated as percentage change of initial TER at time 0 h. TER assays were done on 3 different passages of Caco-2/HT29-MTX cells (n = 3).

### Cytokine measurements

**Figure 1:**
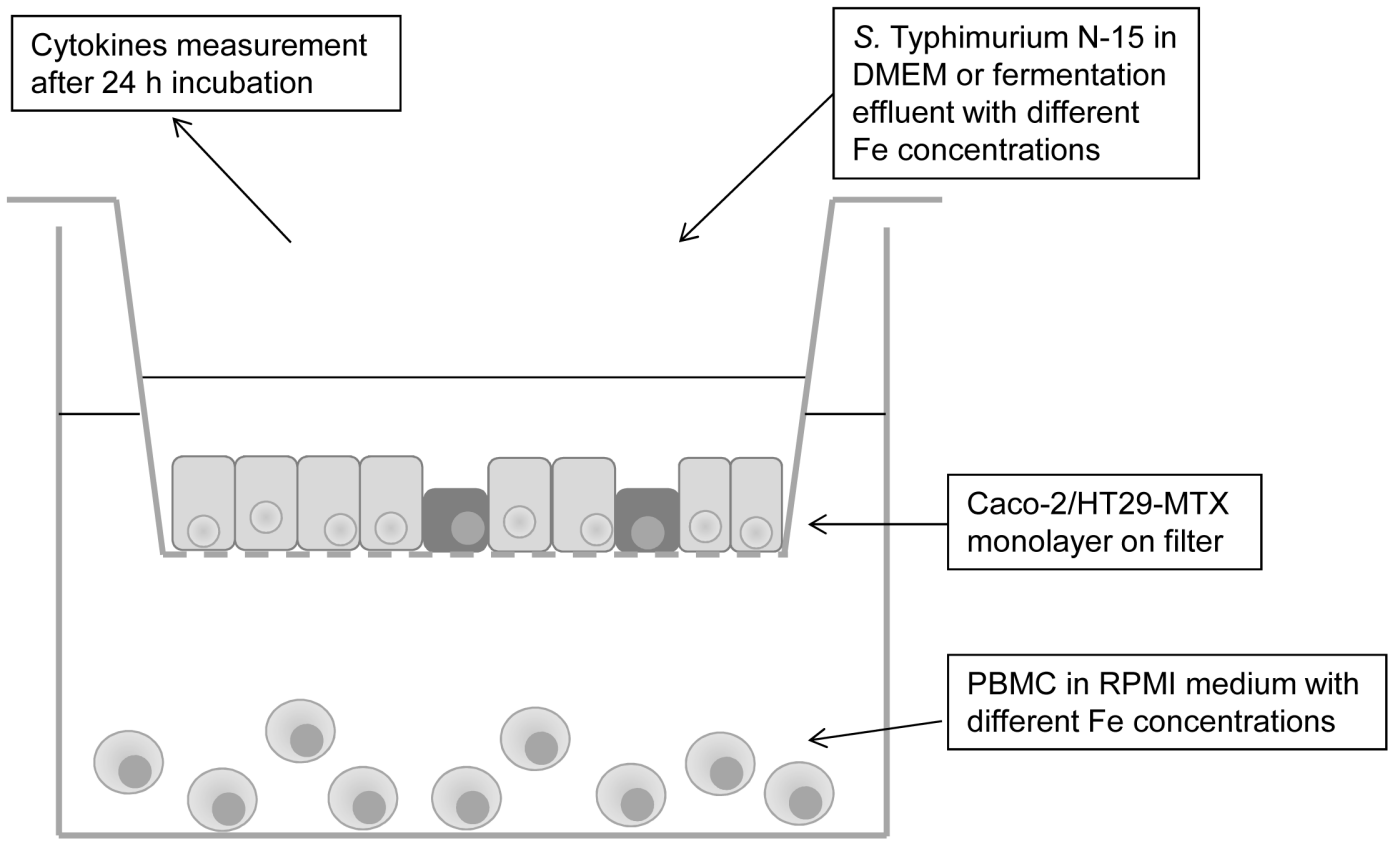
Experimental set up for the analysis of cellular cytokines release. A Caco-2/HT29-MTX co-culture monolayer was grown to confluence on a filter insert of a 24-well plate and freshly collected PBMC were added to the basolateral compartment. *Salmonella* in DMEM or effluent were applied to the apical compartment and incubated for 24 h before cytokines release was measured in the apical supernatant.

Caco-2/HT29-MTX co-cultures from 3 different sequential passages were seeded on cell culture filter membranes (0.45 μm) in 24-well plates (Millipore) as described above and were cultured for 16 days. *S*. Typhimurium N-15 was added to apical wells in DMEM Glutamax or fermentation effluents with different Fe concentrations as described in the adhesion and invasion assays. In the basolateral compartments, 1.2×10^6^ (600 μL) freshly collected PBMC were added in the corresponding Fe concentration to enhance the immune response of cells (**Figure 1**) [41], [42]. PBMC were isolated using Ficoll-Paque PLUS preparation medium (GE Healthcare, Uppsala, Sweden) from a blood sample freshly collected from a healthy volunteer according to manufacturer's instructions. PBMC were then diluted to a concentration of 2×10^6^ cells/mL in Rosewell Park Memorial Institute (RPMI) medium 1640 (Sigma-Aldrich) with different Fe concentrations (normal Fe, low Fe (150 μM 2,2′-dipyridyl), high Fe (14 mg Fe/L, 250 μM Fe)). Cellular exposure to *S*. Typhimurium N-15 in DMEM Glutamax and fermentation effluents was allowed for 24 hours and apical well supernatants were centrifuged and frozen at −80°C until further use. The cytokine release by the Caco-2/HT29-MTX and PBMC co-culture during invasion of *S*. Typhimurium N-15 was evaluated using the 8-plex human cytokine Bioplex kit (Bio-Rad Laboratories, Reinach, Switzerland) according to the manufacturer's instructions. Cytokine release was assessed during invasion in 3 different passages of Caco-2/HT29-MTX (n = 3).

### NF-κB activation in THP1-Blue cells

THP1-Blue cells are human peripheral blood monocytic cells transfected with a NF-κB reporter system and were obtained from Invivogen (LabForce, Nunningen, Switzerland). Upon NF-κB activation, secreted embryonic alkaline phosphatase (SEAP) is expressed and measured in THP1-Blue cells supernatant spectrophotometrically at OD 655 using Quanti-Blue (Invivogen, Labforce). THP1-Blue cells were routinely maintained according to the manufacturer's instructions in RPMI 1640 with 10% heat inactivated FBS, 1% antibiotics (penicillin/streptomycin), 1x non-essential aminoacids, 1x vitamin solution, 2 mM L-glutamine, 1 mM Na-pyruvate (all Invitrogen) and 200 μg/mL zeocin (Invivogen, LabForce). To assess activation of NF-κB under different Fe conditions without pathogen invasion, *E. coli* K-12 lipopolysaccharides (LPS-EK, Invivogen, LabForce) was diluted (10 μg/mL) with RPMI (without any additions) with different Fe concentrations (normal Fe, low Fe (150 μM 2,2′-dipyridyl), high Fe (14 mg Fe/L, 250 μM)). Similarly, *S*. Typhimurium N-15 from a 6 h culture was diluted to 10^7^ cfu/mL with RPMI with different Fe concentrations as described above in adhesion and invasion assays. THP1-Blue cells were seeded at approximately 10^5^ cells/well in 96-well plates. 100 μL LPS-EK RPMI or RPMI containing *S*. Typhimurium N-15 with different Fe concentrations was added to 100 μL cell suspension and incubated for 24 h at 37°C and 5% CO_2_. Supernatants were then centrifuged (1000×g, 10 min), mixed with Quanti-Blue (Invivogen, LabForce) containing 100 μg/mL gentamicin (Sigma-Aldrich) to avoid bacterial growth and incubated again for 24 h at 37°C, 5% CO_2_. Color development was then assessed with a spectrophotometer in duplicate at a wavelength of 655 nm. Each condition was tested in triplicate in two independent experiments (n = 6).

### Statistical analysis

Statistical analysis was done using JMP 8.0 (SAS Institute Inc., Cary, NC, USA). Data were analyzed pairwise using a non-parametric Mann-Whitney test and *P*-values smaller than 0.05 were considered significant. Data are expressed as mean ± standard error of the mean (SEM).

## Results

### Adhesion and invasion capacity of *S*. Typhimurium N-15 is affected by Fe concentration in DMEM

**Figure 2:**
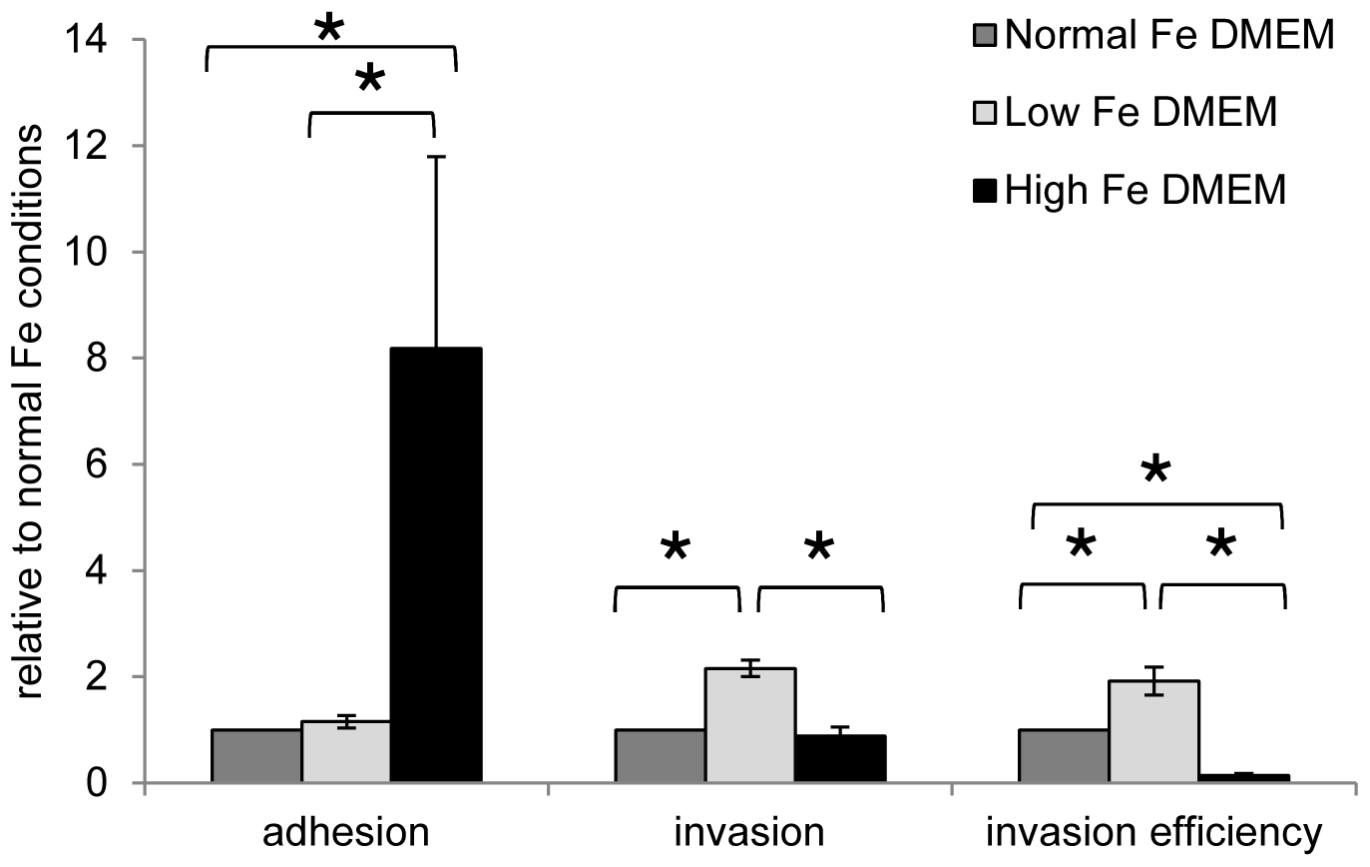
Adhesion, invasion and invasion efficiency of *S*. Typhimurium N-15 under different Fe conditions in DMEM to a Caco-2/HT29-MTX co-culture monolayer. Adhesion, invasion and invasion efficiency ratios are given relative to normal Fe conditions. Values are means ± SEM (n = 3). Values connected with an asterisk (*) are significantly different according to non-parametric Mann-Whitney test (*P*<0.05).

The ability of *S*. Typhimurium N-15 to adhere and invade a Caco-2/HT29-MTX co-culture monolayer was tested under normal Fe conditions, low Fe conditions and high Fe conditions in buffered DMEM with similar *S*. Typhimurium N-15 cell counts of 7.3±0.2 log cfu/mL (mean ± SEM) for all conditions (**Table 1**). Adhesion was strongly increased in a high Fe environment compared to normal or low Fe conditions giving mean values of 193.8±83.9%, 29.0±15.0%, 33.1±16.8%, respectively (Table 1, **Figure 2**). The counts of adhered *S*. Typhimurium N-15 were higher after 1 h incubation compared to the inoculum counts indicating that bacterial growth occured during the test (Table 1). Adhesion was approximately 8 fold increased relative to adhesion in a normal Fe environment (Figure 2). In contrast, invasion was not enhanced by high Fe conditions (1.8±0.2%) compared to normal Fe conditions (2.1±0.4%) resulting in a significantly lower invasion efficiency (ratio of adhered cells to invaded cells) for high Fe conditions. In fact, invasion efficiency decreased during high Fe conditions to only 14.1±4.2% of invasion efficiency during normal Fe conditions (Figure 2). Converse results of adhesion and invasion behavior of *S*. Typhimurium N-15 were obtained under low Fe conditions in the presence of 2,2′- dipyridyl. Adhesion was not significantly changed under low Fe conditions compared to normal Fe conditions, but invasion capacity (4.7±1.1%) was significantly increased compared to normal Fe conditions (2.1±0.4%) and high Fe conditions (1.8±1.6%). This resulted in a two-fold higher invasion efficiency in a Fe restricted environment relative to normal Fe conditions (Figure 2).

**Table 1:** Adhesion, invasion and invasion efficiency of *S*. Typhimurium N-15 under different Fe conditions in DMEM or fermentation effluents.

	Fe condition		
	Normal Fe	Low Fe	High Fe
**DMEM**			
Adhesion [%]^1^	29.0±15.0	33.1±15.8	193.8±83.9
Invasion [%]^1^	2.1±0.4	4.7±1.1*	1.8±0.2**
Invasion efficiency [%]^2^	12.9±6.7	25.1±14.1	2.18±1.6**
**Effluent**			
Adhesion [%]^1^	2.4±0.4	3.3±0.6	4.2±2.4
Invasion [%]^1^	0.005±0.002	0.003±0.001	0.001±0.0005
Invasion efficiency [%]^2^	0.3±0.1	0.1±0.05	0.08±0.03

Values are means ± SEM (n = 5). Values with an asterisk (*) are significantly different from normal Fe conditions, values with double asterisk (**) are significantly different from low Fe conditions, within the same parameter, Mann-Whitney test (*P*<0.05).

1 Expressed as percentage of inoculum *Salmonella* concentration; ^2^Expressed as percentage of adhered to invaded *Salmonella*.

### The gut microbiota changes the impact of Fe on *S*. Typhimurium N-15 adhesion and invasion

**Figure 3:**
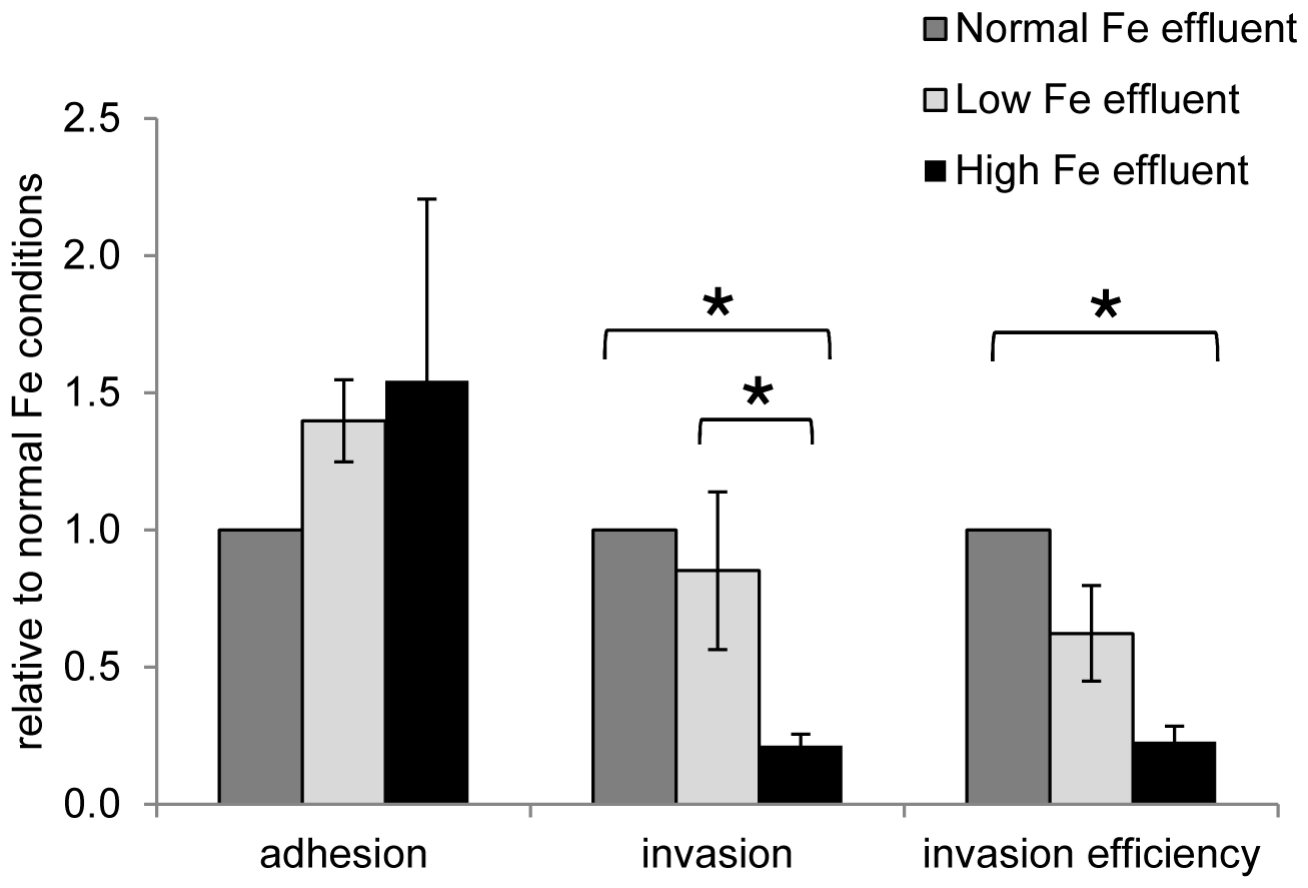
Adhesion, invasion and invasion efficiency of *S*. Typhimurium N-15 under different Fe conditions in effluent to a Caco-2/HT29-MTX co-culture monolayer. Adhesion, invasion and invasion efficiency ratios are given relative to normal Fe conditions. Values are means ± SEM (n = 5). Values connected with an asterisk (*) are significantly different according to non-parametric Mann-Whitney test (*P*<0.05).

Adhesion and invasion of *S*. Typhimurium N-15 was also tested in the presence of a complex gut microbiota sample derived from continuous *in vitro* colonic fermentations used to investigate the impact of different Fe concentrations on the commensal gut microbiota [36]. Effluents were mixed with fresh *S*. Typhimurium N-15 cells to yield the same concentration of viable *S*. Typhimurium N-15 in normal Fe, low Fe and high Fe effluents of 7.26±0.19 log cfu/mL. In the presence of commensal microbiota and metabolites, adhesion of *S*. Typhimurium N-15 was 12-fold decreased and invasion was 428-fold decreased in normal Fe effluents compared to adhesion and invasion in normal Fe DMEM (Table 1). Relative to normal Fe conditions, invasion and invasion efficiency were significantly impaired in high Fe effluents compared to normal Fe conditions (**Figure 3**). Unlike in DMEM, low Fe effluent had no impact on invasion and invasion efficiency of *S*. Typhimurium N-15 compared to normal Fe conditions.

### Monolayer integrity is similarly affected by *S*. Typhimurium N-15 under different Fe conditions and incubation with effluent causes slight cell toxicity

**Figure 4:**
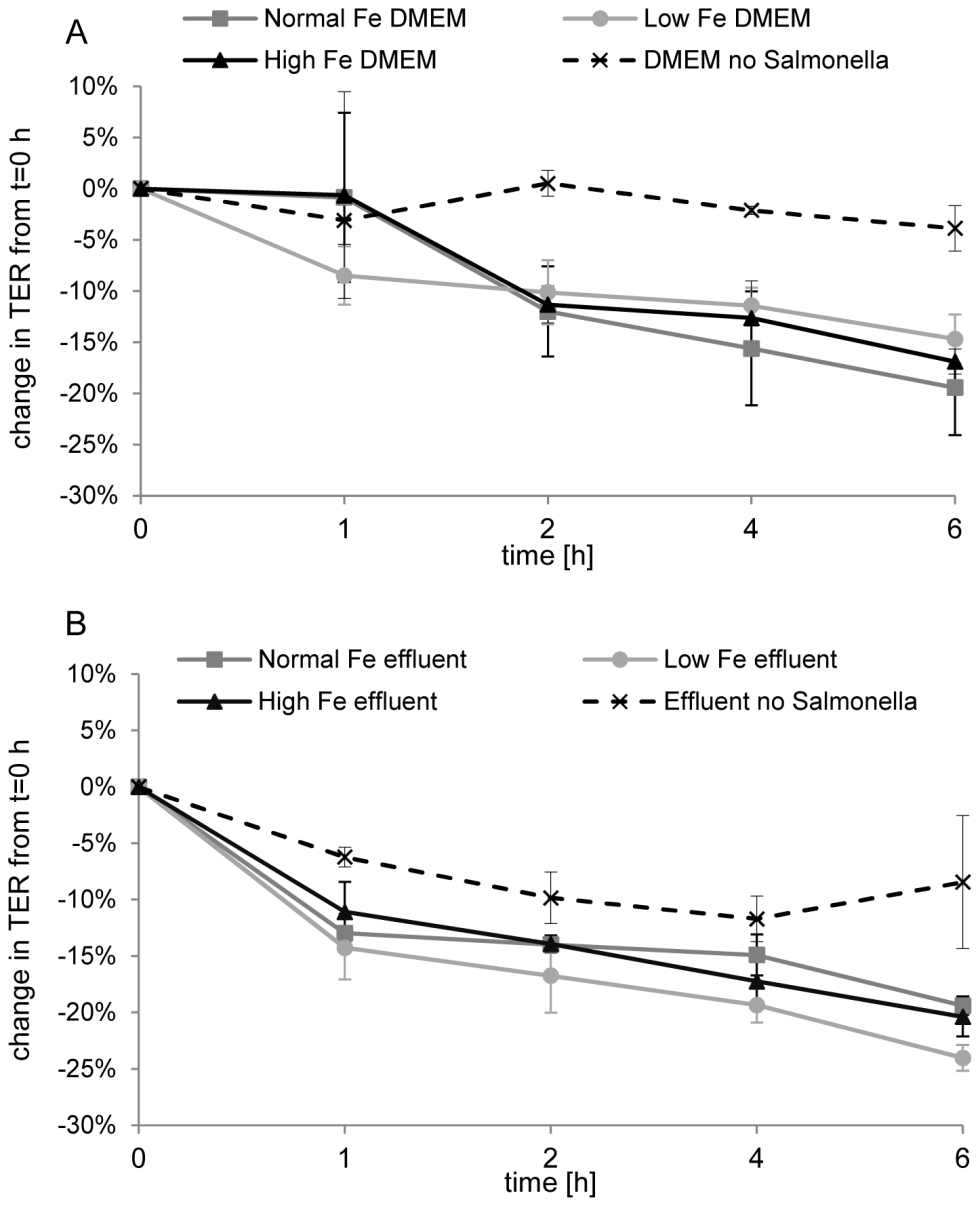
Epithelial integrity assessment during *Salmonella* invasion. TER across Caco-2/HT29-MTX co-culture monolayers during invasion of *S*. Typhimurium N-15 in DMEM (A) or in the presence of a complex commensal microbiota (B, effluent) under different Fe conditions. Change in TER values as percentage of initial TER at time 0 h after 1, 2, 4, and 6 h of incubation with DMEM or fermentation effluent containing *S*. Typhimurium N-15. Values are means ± SEM (n = 3; High Fe effluent, n = 2).

The integrity of the Caco-2/HT29-MTX monolayer co-culture in the presence of *S*. Typhimurium N-15 in DMEM (**Figure 4A**) and effluents (**Figure 4B**) with different Fe conditions was evaluated by the transepithelial resistance across the monolayer after t = 0, 1, 2, 4 and 6 h of incubation. The percentage decrease in monolayer integrity after application of *S*. Typhimurium N-15 in DMEM was similar over the time course of 6 h for all Fe concentrations tested (Figure 4A). However, the presence of *S*. Typhimurium N-15 decreased the cellular integrity significantly in all Fe conditions (normal Fe DMEM, −19.5±4.6%; low Fe DMEM, −14.7±2.4%; high Fe DMEM, −16.9±1.2%) compared to DMEM without *S*. Typhimurium N-15 (−3.9±2.2%) after 6 h. In the presence of a commensal microbiota, monolayer integrity was also strongly affected. Cell integrity decreased similarly for different Fe concentrations in effluents containing *S*. Typhimurium N-15. However, fermentation effluents containing a complex microbiota without *S*. Typhimurium N-15 also exhibited decreased monolayer integrity after 6 h (loss of TER of −8.5±5.9%). This suggests that complex fermentation effluents alone can affect monolayer integrity. Therefore, the cytotoxic effects of effluents with *Salmonella* were tested by measuring lactate dehydrogenase release by using the Cytotox assay (Promega) after 1 h incubation. LDH release into cellular supernatant after 1 h incubation with normal, low and high Fe effluent containing *S*. Typhimurium N-15 was 7.3±0.8%, 10.9±2.0% and 10.7±1.0%, respectively, of maximum LDH release (defined as LDH release after 1 h invasion of pure *S*. Typhimurium N-15 culture in normal Fe DMEM). Only effluents with high Fe concentration caused a slightly higher cytotoxicity (p = 0.0495) compared to normal Fe effluent.

### Cellular immune response to *S*. Typhimurium N-15 invasion is decreased in the presence of a commensal microbiota and high Fe conditions

**Figure 5:**
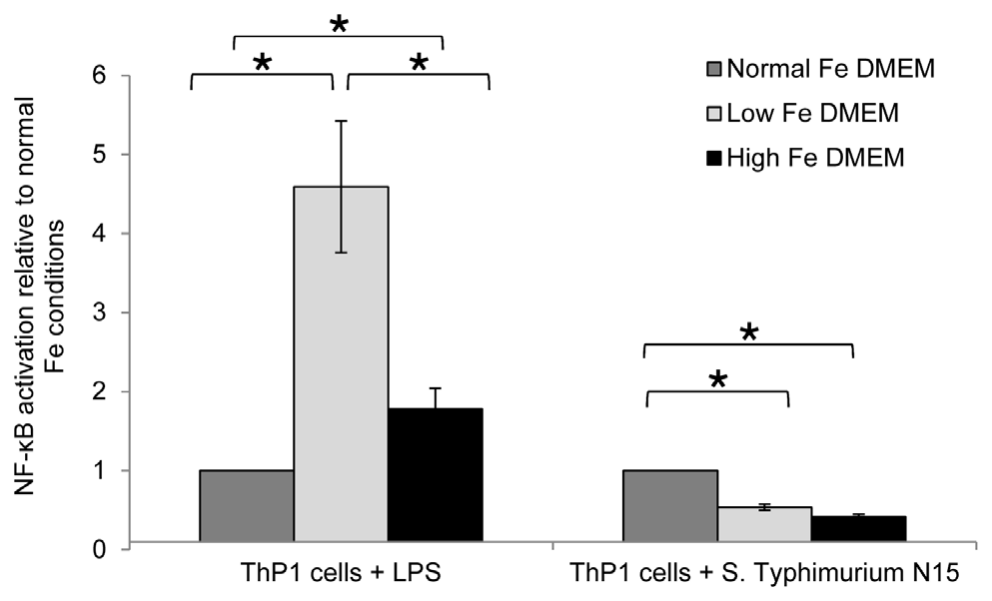
Inflammatory response in THP1-Blue cells under different Fe conditions. THP1-Blue cells with an NF-κB reporter were incubated for 24 h with either LPS-EK or *S*. Typhimurium N-15 and NF-κB activation was determined by measuring the SEAP activity in the cell supernatant spectrophotometrically (QUANTI-Blue). Activation of NF-κB is given relative to NF-κB activation under normal Fe conditions. Values are means ± SEM (n = 6). Values connected with an asterisk (*) are significantly different according to non-parametric Mann-Whitney test (*P*<0.05).

The cellular immune response during infection with *S*. Typhimurium N-15 under different Fe concentrations with and without commensal gut microbiota was assessed using the Bioplex kit (BioRad) as well as THP1-Blue cells transfected with a NF-κB-inducible reporter system. IL-1β, IL-6, IL-8 and TNF-α concentrations were measured in cellular supernatants after 24 h incubation with *S*. Typhimurium N-15 under different Fe conditions in DMEM and fermentation effluents (**Table 2**). Invasion under effluents with normal Fe conditions caused lower IL-1β and lower IL-6 release in the presence of a commensal microbiota compared to invasion in DMEM. Interestingly, cytokines release during *S*. Typhimurium N-15 infection in high Fe DMEM was significantly lower for all tested cytokines compared to normal Fe DMEM. Moreover, IL-8 release was strongly induced during infection under low Fe DMEM. Monolayer infection of *S*. Typhimurium N-15 in the presence of low Fe or high Fe fermentation effluents did not change cytokines expression of IL-1β, IL-6 or IL-8 compared to normal Fe effluent. However, TNF-α expression was strongly decreased under high Fe effluent compared to normal Fe effluent. Also *S*. Typhimurium N-15 invasion under high and low Fe RPMI medium decreased NF-κB activation in THP1-Blue cells compared to invasion under normal Fe conditions (**Figure 5**). In order to test the immune response without an actively invading pathogen, THP1-Blue cells were challenged with LPS-EK under different Fe conditions in RPMI. NF-κB activation was assessed by measuring the SEAP activity in the cell supernatant (Figure 5). Interestingly, a low Fe concentration and a high Fe concentration significantly increased NF-κB activation in the presence of LPS-EK compared to normal Fe conditions.

**Table 2:** Cytokine secretion of Caco-2/HT29-MTX monolayers co-cultured with PBMC during 24 h infection with *S*. Typhimurium N-15 in DMEM or fermentation effluent under different Fe conditions.

	Fe condition		
	Normal Fe	Low Fe	High Fe
**DMEM**			
IL-1β [pg/mL]	198.82±53.44	245.17±40.82	6.51±2.09*
IL-6 [pg/mL]	8.01±2.26	9.53±1.66	0.56±0.25*
IL-8 [pg/mL]	327.14±80.66	968.42±103.54*	94.28±21.31*
TNF-α [pg/mL]	24.23±6.91	36.89±4.61	2.41±2.03*
**Effluent**			
IL-1β [pg/mL]	5.04±1.24	10.07±4.08	6.89±4.22
IL-6 [pg/mL]	2.70±0.52	2.22±0.27	9.72±5.59
IL-8 [pg/mL]	228.77±22.74	383.15±152.96	170.36±61.73
TNF-α [pg/mL]	30.55±6.93	11.65±2.74*	6.47±0*

Values are means ± SEM (n = 3). Values with an asterisk (*) are significantly different from normal Fe conditions within the same cytokine, Mann-Whitney test (*P*<0.05).

## Discussion

Combined models using *in vitro* fermentation effluents with a complex gut microbiota and intestinal cells present a valuable platform for investigating the complex microbes-microbes and microbes-host interactions in the gut lumen [22], [27], [43]. The approach of using intestinal cellular co-cultures simulating more closely the intestinal epithelial layer has been shown to mimic *in vivo* conditions for Fe absorption, bacterial adhesion and immune response [37], [44], [45], [46], [47]. Moreover, when investigating the impact of *Salmonella* invasion in the presence of a commensal microbiota, the environmental parameters such as bacterial composition and metabolites production might play an important role. Therefore, we used a gut microbial consortium impacted by different Fe conditions, encountered during Fe deficiency and Fe supplementation in the gut lumen, derived from a continuous *in vitro* colonic fermentation model inoculated with immobilized child fecal microbiota [36] and investigated the influence on *Salmonella* adhesion and invasion. The changes in the microbiota due to low Fe availability during the continuous *in vitro* fermentation were very distinct and resulted in a much lower propionate and butyrate concentration in low Fe effluent (72±5 mM acetate, 4±1 mM propionate, 8±2 mM butyrate) compared to normal Fe effluent (70±4 mM, 8±1 mM, 44±3 mM) or high Fe effluent (61±4 mM, 10±1 mM, 51±2 mM) [36]. Moreover, *Enterobacteriaceae*, *Lactobacillus/Pediococcus/Leuconostoc* spp. and *Bifidobacterium* spp. dominated in low Fe effluent compared to normal or high Fe effluent while other bacterial species such as *Bacteroides* spp. and *Roseburia* spp./*E. rectale* were decreased.

Adhesion rates in our study under normal Fe DMEM (29.0±15.0%) are comparable with other studies investigating the adhesion of *Salmonella* to mucus secreting epithelial cells [13], [22], [39]. Moreover in this study, a very distinct 8-fold increase of adhesion under very high Fe conditions was observed which has also been reported previously by Kortman *et al*. [13]. In fact, adhered *S*. Typhimurium N-15 counts even exceeded the inoculum concentration suggesting that Fe promoted *S*. Typhimurium N-15 growth during the assay carried out under high Fe conditions and induced bacterial adhesion. Unexpectedly, invasion of *S*. Typhimurium N-15 was not similarly promoted by high Fe conditions as adhesion. However, several *Salmonella* virulence factors are positively regulated by Fe, such as type III secretion system [8], [10], [11]. It has been reported that Fe concentrations up to 10 μM Fe led to an increased invasion capacity of *Salmonella in vitro* into Caco-2 cells, whereas a further increase in Fe concentration did not further increase invasion [12], [13]. The Fe concentration used in our study was aimed to mimic Fe concentrations in the child gut lumen during Fe supplementation [36] assuming that a daily treatment of 60 mg Fe with a total chyme volume of 1 to 2 liters per day would result in an average Fe concentration of 500 to 1000 μM Fe. In contrast, most studies applied much lower Fe concentrations when investigating the influence of Fe on invasion of *Salmonella*. Hence, the Fe concentration in high Fe DMEM for our study was set to 250 μM Fe which allowed both growth of *Salmonella* and epithelial cells. Therefore, the hypothesis that invasion efficiency of *S*. Typhimurium N-15 would be increased due to high Fe concentrations in the gut lumen during Fe supplementation via stimulation of virulence factors was not supported by the results of this study.

The presence of the Fe chelator 2,2′-dipyridyl did not alter the adhesion pattern of *S*. Typhimurium N-15 but significantly increased invasion. In a previous study, *Salmonella* virulence genes encoding for intracellular survival were upregulated in the presence of 2,2′-dipyridyl mimicking an Fe deficient environment encountered intracellularly during inflammation [48].

Research in the past years has highlighted the role of the gut microbiota in enteric pathogen infection. Barrier function is probably mediated by the gut microbiota composition and diversity as well as metabolic activity to generate an environment hostile to *Salmonella,* where adhesion sites and nutritional niches are occupied, inhibitory metabolites are produced and the immune defenses of the host are stimulated [16]. In our study we have observed a significant and marked decrease in *S*. Typhimurium N-15 adhesion (12-fold decrease) and invasion (428-fold decrease) in fermentation effluents compared to adhesion and invasion in DMEM. A decreased adhesion and invasion in the presence of a commensal microbiota has also been observed by Zihler *et al*. in a similar *in vitro* gut fermentation – cell model [22]. *In vivo* animal studies revealed that a dysbiosis of the gut microbiota caused by preceding antibiotic treatment led to much higher *Salmonella* colonization and gut epithelial pathology scores after *Salmonella* infection [16], [49], [50]. We then hypothesized that dysbiosis of the gut microbiota caused by different Fe concentrations in the gut during Fe deficiency and Fe supplementation impacts *Salmonella* adhesion and invasion besides the sole influence of Fe as investigated in DMEM. We observed that low Fe conditions decreased butyrate (−84%) and propionate (−55%) and increased acetate concentration in *in vitro* gut fermentation effluents compared to normal Fe conditions, while lactobacilli and bifidobacteria numbers were strongly increased [36]. Unexpectedly, low Fe fermentation effluents had no impact on *S*. Typhimurium N-15 adhesion or invasion whereas invasion was increased with low Fe DMEM. Increased *Salmonella* invasion with low butyrate and propionate and high acetate has been reported in previous studies *in vitro* [17] and in chicken [19]. On the other hand, high levels of lactobacilli and bifidobacteria were shown to have protective effects against *Salmonella* invasion *in vitro* [22], [24], [51] and *in vivo* in chicken [52] and mice [25]. Therefore, the effects of low Fe effluents on *S*. Typhimurium N-15 adhesion and invasion are complex, being the result of combined factors such as commensal bacterial composition and metabolic activity.

In high Fe effluent, the gut microbiota composition and metabolites profile was similar to normal Fe effluent [36] whereas *S*. Typhimurium N-15 invasion efficiency was decreased to approximately 20% of the invasion efficiency under normal Fe effluent (Figure 3). A similar effect was observed in high Fe DMEM without a commensal microbiota suggesting that impairment of invasion efficiency may be a direct effect of the high Fe concentrations encountered in the child gut lumen during oral Fe supplementation. Moreover, *Salmonella* growth and adhesion was not promoted in high Fe effluent as observed in high Fe DMEM. This may be due to the strong competition for nutrients within a complex bacterial ecosystem preventing growth of *Salmonella* although Fe is highly abundant or due to the capability of other bacteria to scavenge Fe from the environment reducing the amount of Fe needed for *Salmonella* growth promotion.

*In vivo* studies investigating the impact of Fe on *Salmonella* invasion have reported contradictory results. Mice treated with the Fe chelator deferoxamine and infected with *S*. Typhimurium had much higher concentrations of the pathogen in liver and spleen compared to mice not pretreated with deferoxamine [14] while another study reported that highly Fe deficient mice had a higher survival after *Salmonella* infection compared to mice fed a normal Fe diet [53]. On the other hand, patients suffering from Fe overload are reported to be at a higher risk of infection [15], [54]. These contradictory findings may be explained by the fact that Fe not only plays an important role in pathogen invasion but also changes the immune defense of the host [3]. The cellular immune defense was assessed by cytokine release into the cellular supernatant of a Caco-2/HT29-MTX co-culture with PBMC added to better mimic the inflammation response [41], [42]. Interestingly, *S*. Typhimurium invasion under high Fe conditions in DMEM resulted in a significantly lower cytokines production compared to normal Fe conditions although absolute invasion was not significantly lower while in THP1 cells also a decreased NF-κB induction was observed. These findings are in agreement with previous studies where Fe overload led to a decrease of especially TNF-α release in general [3], [15] and during *Salmonella* invasion under high Fe conditions [5]. This implements negative regulatory effects of high Fe conditions on cellular immune functions and thus might impair effective combat of invading *S*. Typhimurium N-15 which may contribute to exacerbated infections in patients suffering from Fe overload [15], [54]. Interestingly, in the presence of a commensal microbiota the generally decreased inflammation response under high Fe conditions was only partially observed, likely due to immunoregulatory functions of the gut microbiota and its metabolites [27], [41], [55]. However, TNF-α was decreased in both high Fe effluents and in high Fe DMEM supporting a direct down regulation effect of high Fe concentration on TNF-α as previously observed [3]. Intracellular Fe levels were not measured in this experiment, limiting the ability to conclude if intra- or extracellular Fe may have contributed to the observed results as intracellular Fe also influences cytokine release *in vivo* [56], [57]. The presence of a microbial environment decreased the pro-inflammatory cytokines release of IL-1β and IL-6 during *S*. Typhimurium N-15 invasion in fermentation effluents and lowered invasion capacity compared to DMEM. It has been shown previously that the presence of commensal gut bacteria, such as bifidobacteria, can decrease the production of several pro-inflammatory cytokines in epithelial cells [26], [27]. Interestingly, when THP1-Blue cells were challenged under different Fe conditions with bacterial LPS, only interacting with NF-κB inducing TLR receptors on the cell surface, cellular immune response (NF-κB activation) was strongly increased under low and high Fe conditions compared to normal Fe conditions. This indicates that mostly intracellular *Salmonella* are responsible for the observed immune responses under different Fe conditions.

Epithelial integrity (TER) was decreased similarly and independent of Fe concentration by *S*. Typhimurium N-15 in DMEM and fermentation effluents, in agreement with previous studies where the impact of S. Typhimurium on cellular integrity was measured [13], [22]. Cell toxicity of all effluents was similar. These results indicate that cellular viability was similarly influenced by different Fe conditions in fermentation effluents.

Our *in vitro* study combining *in vitro* gut fermentation and cell models showed that the gut microbial environment strongly attenuated *Salmonella* invasion while decreasing inflammatory response. Furthermore, the gut luminal Fe conditions encountered during Fe supplementation did not increase *in vitro Salmonella* invasion as previously assumed. However, cellular immune response was also strongly decreased under high Fe conditions which could weaken the host response against *Salmonella*. Moreover, the increased risk of infection during Fe supplementation with other pathogens, such as malaria and *Mycobacterium tuberculosis*, could generate an inflammatory preset of the host which in turn might facilitate co-infection with enteropathogens [33], [58]. This would be especially problematic in regions with a high infectious disease burden, such as in developing countries. Therefore, further *in vivo* investigations are needed also considering the Fe status and the inflammatory preset of the host as well as the occurrence of other infections to study the effect of Fe supplementation on pathogen invasion. Moreover, the consequences of the observed results from our study on host health can only be fully evaluated *in vivo*. Nevertheless, our *in vitro* studies have highlighted the complexity of Fe effects for the microbial ecosystem of the gut and the importance of models closely mimicking host conditions, such as the commensal microbiota, when studying gastrointestinal pathogens and host interactions.
